# Low Frequency Ultrasound With Injection of NMO-IgG and Complement Produces Lesions Different From Experimental Autoimmune Encephalomyelitis Mice

**DOI:** 10.3389/fimmu.2021.727750

**Published:** 2021-10-14

**Authors:** Weiwei Xiang, Chong Xie, Jiaying Luo, Wei Zhang, Xinxin Zhao, Hong Yang, Yu Cai, Jie Ding, Yishu Wang, Yong Hao, Ying Zhang, Yangtai Guan

**Affiliations:** ^1^ Department of Neurology, Ren Ji Hospital, Shanghai Jiao Tong University School of Medicine, Shanghai, China; ^2^ Department of Ultrasound in Medicine, Shanghai Jiao Tong University Affiliated Sixth People’s Hospital, Shanghai, China; ^3^ Department of Radiology, Ren Ji Hospital, Shanghai Jiao Tong University School of Medicine, Shanghai, China; ^4^ Department of Neurology, The First Rehabilitation Hospital of Shanghai, Tongji University School of Medicine, Shanghai, China

**Keywords:** neuromyelitis optica, mouse, Aquaporin-4, blood-brain barrier, low-frequency ultrasound

## Abstract

Neuromyelitis optica spectrum disorder (NMOSD), a relapsing autoimmune disease of the central nervous system, mainly targets the optic nerve and spinal cord. To date, all attempts at the establishment of NMOSD animal models have been based on neuromyelitis optica immunoglobulin G antibody (NMO-IgG) and mimic the disease in part. To solve this problem, we developed a rodent model by opening the blood-brain barrier (BBB) with low frequency ultrasound, followed by injection of NMO-IgG from NMOSD patients and complement to mice suffering pre-existing neuroinflammation produced by experimental autoimmune encephalomyelitis (EAE). In this study, we showed that ultrasound with NMO-IgG and complement caused marked inflammation and demyelination of both spinal cords and optic nerves compared to blank control group, as well as glial fibrillary acidic protein (GFAP) and aquaporin-4 (AQP4) loss of spinal cords and optic nerves compared to EAE mice and EAE mice with only BBB opening. In addition, magnetic resonance imaging (MRI) revealed optic neuritis with spinal cord lesions. We further demonstrated eye segregation defects in the dorsal lateral geniculate nucleus (dLGN) of these NMOSD mice.

## Introduction

Neuromyelitis optica spectrum disorder (NMOSD), a relapsing autoimmune disease of the central nervous system (CNS), mainly targets the optic nerve and spinal cord. The incidence of NMOSD is approximately 5 per 100,000 people and mainly affects Asian people (1–3). NMOSD has a high disability rate, which seriously affects the quality of life and imposes a huge burden on society. Aquaporin 4 (AQP4)-specific antibodies (AQP4-abs) are found in the majority of patients with NMOSD and are termed neuromyelitis optica immunoglobulin G antibodies (NMO-IgG). These antibodies are highly pathogenic when they are applied together with complement *in vitro* and *in vivo* (4–7).

To date, all attempts at the establishment of the NMOSD model are based on NMO-IgG and mimic the disease in part. However, all previous animal models of NMOSD were limited by the tremendous demand for systemic NMO-IgG (intraperitoneal/intravenous injection) or problems posed by direct injection (intracerebral/perichiasmal/intravitreal injection). Our previous study established an animal model of NMOSD by additionally compromising the blood-brain barrier (BBB) with low-frequency ultrasound to enable efficient access for NMO-IgG and complement to enter the CNS of experimental autoimmune encephalomyelitis (EAE) mice. Our NMOSD model was able to induce the pathogenesis of NMOSD with an amount of NMO-IgG as low as 100 μg (8). We have also successfully demonstrated inflammatory demyelination concomitant with the loss of AQP4 and glial fibrillary acidic protein (GFAP) expression in the spinal cord, brain and optic nerve. However, NMO-IgG was injected into EAE mice, which had already exhibited myelitis and optic neuritis. No EAE control group was included in our previous study. Therefore, an EAE control group should be included. In addition, the core clinical characteristics induced by NMO-IgG were not fully dissected with magnetic resonance imaging (MRI), one of the most important methods for the diagnosis of NMOSD patients as well as a few mouse EAE models (9–13).

Furthermore, visual pathway damage, one of the key features of optic neuritis, was also commonly observed in NMOSD patients (14). In the mammalian visual system, retinal ganglion cell inputs from each eye, initially intermixed within the dLGN, become segregated during development (15). When unilateral visual input is deprived, the projection from the unaffected side is greatly expanded into territory normally belonging to the blocked side, and the projection from the inactive side is substantially reduced (16). This can be reflected by eye segregation defects in the dorsal lateral geniculate nucleus (dLGN). Thus, in addition to MRI, we took advantage of the labeling of retinal ganglion cell (RGC) projections to examine injury along the visual pathway and defects in eye-specific segregation in our NMOSD model.

The aim of this study was to compare the clinical and pathological manifestations of an NMOSD model established by low-frequency ultrasound in EAE mice. We tested whether low-frequency ultrasound applied to EAE mice can further compromise BBB integrity and boost clinical disease. We also investigated the imaging features of the NMOSD model.

## Materials and Methods

### Mice

C57BL/6 WT mice (6–8 weeks old, female) were used. All animal protocols were reviewed and approved by the animal care and use committees of Renji Hospital Affiliated to Shanghai Jiaotong University School of Medicine.

### Preparation of NMO-IgG and Complement

NMO-IgG and complement were obtained as described previously (8, 17). Briefly, serum was obtained from NMOSD patients seropositive for NMO-IgG. NMO-IgG was purified from sera using the Melon Gel IgG Purification Kit (Thermo Fisher Scientific). Complement was collected from human volunteers. Blood samples were obtained and prepared as described previously (8). Informed consent was obtained from all subjects. The study protocol was approved by the Ethics Committee of the Renji Hospital Affiliated to Shanghai Jiaotong University School of Medicine.

### NMOSD Model Induction

First, mice received a subcutaneous immunization of 200 μg of myelin oligodendrocyte glycoprotein (MOG)_35–55_ (GL Biochem) emulsified in complete Freund’s adjuvant containing 4 mg/ml Mycobacterium tuberculosis (H37Ra; Difco Laboratories). On Day 0 and 2 postimmunization (p.i.), 200 ng of pertussis toxin (List Biological) was injected intraperitoneally. Then, on Day 15 p.i., the BBB was opened by microbubble-enhanced low-frequency ultrasound (MELFUS) (8). Briefly, mice were anesthetized and their hair on the targeted area was removed with depilatory cream. Microbubble solution (12 ul/g, body weight) was injected intravenously followed by ultrasound treatment for 2 minutes. 100 μL NMO-IgG (the concentration of IgG was 1.09 mg/ml) and 100 μL human complement were injected through the tail vein of each mouse 2 min after the opening of the BBB. In addition to the NMOSD group, the EAE group (mice induced for EAE), EAE + BBB group (EAE mice with BBB opened by MELFUS but received no NMO-IgG) and control group (female mice without receiving the emulsion and pertussis toxin) were also studied.

### BBB Permeability Assay

At 1/60/120/180/240/300 min after the sonication procedure, 2% Evans Blue (EB) dye solution (5 ml/kg) was injected through the tail vein. Mice were sacrificed and received intracardiac perfusion two hours after EB injection. Brains were harvested, and sectioned in 20 um for fluorescence imaging. For the EB concentration measurement, brains were weighed and homogenized in 50% trichloroacetic acid solution. The homogenized samples were centrifuged at 12,000 x g for 20 minutes. The supernatant was harvested and diluted with ethanol. The amount of EB was quantified at Ex 620 nm and Em 680 nm by a spectrophotometer.

### Histology Staining and Analysis

The mice were sacrificed on Day 24 p.i. and perfused with PBS. Spinal cords, optic nerves and brains were harvested for pathological assessment. H&E staining and Luxol fast blue (LFB) staining were conducted as previously described (8, 17). The percentage of infiltration was calculated as the number of inflammatory cells in different groups relative to control group, while the demyelination severity was expressed as the relative percentage of reduced blue area to the control group. The observers were blinded to the experimental groups. For immunohistochemistry, spinal cords, optic nerves and brains were fixed, sliced and stained with primary antibodies against GFAP (1:1000; Abcam), AQP4 (1:1000; Abcam), NeuN (1:300, Abcam), occludin (1:200, invitrogen) and claudin-5 (1:200, invitrogen) overnight. An appropriate secondary antibody (Thermo Fisher Scientific, USA) was applied for 1h at room temperature. The results were visualized by fluorescence microscopy and analyzed by ImageJ.

### Magnetic Resonance Imaging Scanning

Spinal cord, optic nerve and brain MRI examinations were performed using a 7-T small-animal MRI instrument, a 30-cm horizontal bore magnet, and a BioSpec Avance III spectrometer (Bruker Daltonics). T2-weighted images were acquired. DWI: A 3-dimensional T2-weighted fast spin echo sequence was employed for structural imaging with the following parameters: field of view (FOV) = 15.0 × 15.0 ×3.0 mm, resolution = 100 × 100 × 150 μm, repetition time (TR) = 3000 ms, effective echo time = 48 ms, number of signal averages = 1, and imaging time = 15 min.

### Detection of Eye-Specific Projections to the dLGN

Mice were anesthetized and 1.5 μL of fluorescent dye was injected intravenously using a pulled glass pipette (tip diameter ~5 μm) (Alexa 488 (green) for the left eye and Alexa 555 (red) for the right eye). 48 hours after surgery, animals were sacrificed and received intracardiac perfusion. Brains were harvested, fixed in 4% paraformaldehyde (PFA) for 12 h and mounted in 3% agarose. Brain slices (100um) were sectioned coronally using a vibratome (1000VT, Leica). Three slices with the largest ipsilateral projection area were collected for imaging and analysis.

### Statistical Analysis

Statistical analysis was performed using GraphPad Prism 5 (GraphPad Software, San Diego, CA). Clinical scores were analyzed by calculating the area under the curve for each mouse over the clinical period of the experiment. Data were presented as mean ± SEM. Differences between two groups were compared by two-tailed unpaired t test, while differences among three groups were compared by one-way ANOVA. A value of p < 0.05 was considered statistically significant.

## Results

### Low-Frequency Ultrasound Caused BBB Opening

**Figure 1:**
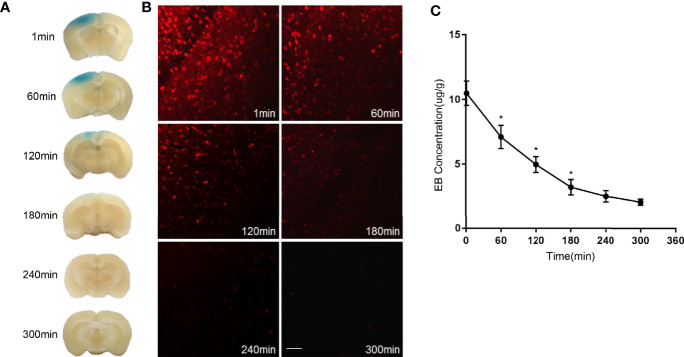
Evans Blue (EB) dye extravasation due to BBB opening. **(A)** Representative images of the mouse brain harvested two hours after EB injection. **(B)** Fluorescence images of EB dye extravasation in the mouse brain. **(C)** EB concentration measurement in the mouse brain. Scale bar, 100µm. The experiment was repeated twice, with similar results. Data were presented as the mean±SEM; *P < 0.05 vs group at the former time points; n=6 in each group. LSD-t test was used.

We first tested whether the BBB could be opened by low-frequency ultrasound and microbubbles. Mice receiving MELFUS manipulation were immediately intravenously injected with EB, a special dye that cannot cross the BBB when it is closed. Photographs of the mouse brain were harvested 2 h after EB injection (**Figure 1A**). Spots with slight blue coloration in the brain due to EB extravasation were visible, indicating that the BBB was successfully opened. Similarly, fluorescence images also showed EB dye extravasation into the mouse brain (**Figure 1B**). We found that the opening of the BBB decreased over time: at 1 min after the sonication procedure, the maximum level of BBB opening was observed; at 60 min, 120 min and 180 min, both the blue coloration and red fluorescence faded, in accordance with the measurement of EB concentration in the mouse brain (**Figure 1C**); at 180 min after the sonication procedure, the BBB was almost closed. From the above results, we confirmed that the BBB was opened by low-frequency ultrasound with microbubbles.

**Figure 2:**
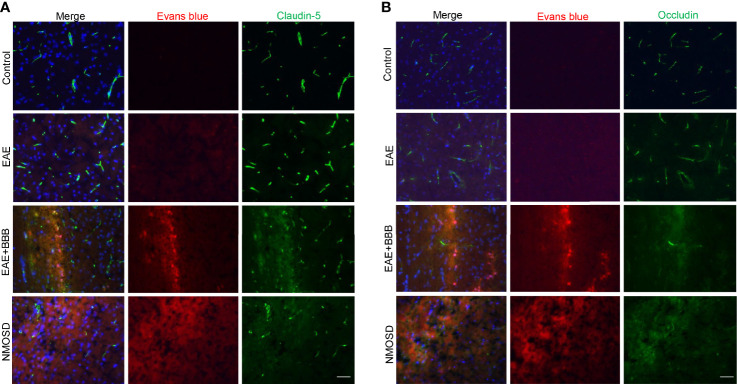
Loss of tight junction components following low-frequency ultrasound. **(A, B)** Immunofluorescence revealed loss of the tight junction protein, occludin and claudin-5, following low-frequency ultrasound.

Whether tight junction components, such as occludin and claudin 5, would be affected by low-frequency ultrasound was further investigated. EB dye was used to indicate the site of BBB opening caused by low-frequency of ultrasound, and co-immunostaining of occludin or claudin-5 was carried out to indicate the change of tight junction components in this site. As shown in (**Figures 2A, B**), low-frequency ultrasound caused further BBB opening, which is indicated by lower expression levels of occludin and claudin-5 in the NMOSD group, compared to the EAE group. These results suggest low-frequency ultrasound additionally compromised the integrity of an already disturbed BBB in EAE mice.

### Significant Changes in EAE Score After NMO-IgG Injection

**Figure 3:**
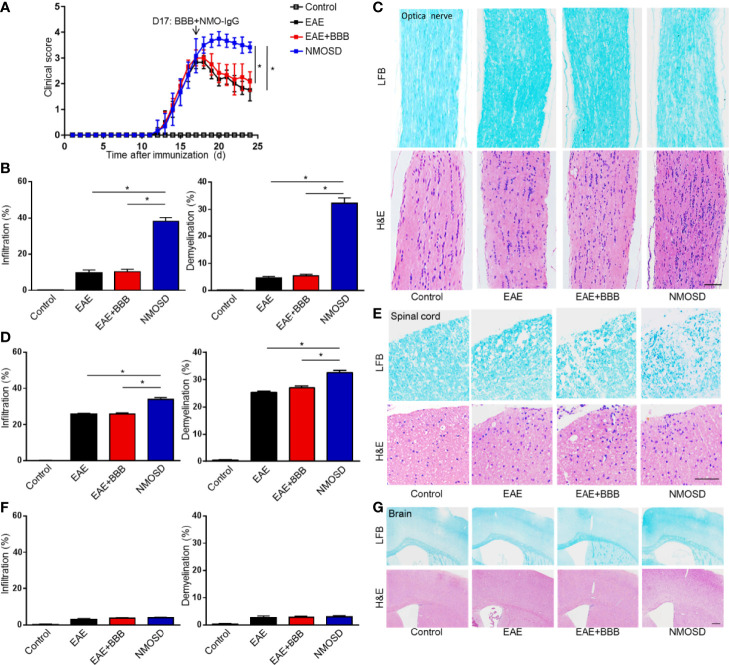
Severe symptom and marked inflammatory demyelination in NMOSD model. **(A)** The EAE scores of mice peaked on day 17 after immunization: the BBB was opened and NMO-lgG plus complement was injected on day 17; **(B)** Statistics of immune cell infiltration, reflected by H&E staining, and demyelination level, reflected by LFB staining, of the optic nerve; **(C)** H&E staining showed extensive inflammation in the optic nerve of NMOSD mice. LFB staining depicted demyelination in the optic nerve of NMOSD mice. Scale bar=50µm; **(D)** Statistics of immune cell infiltration, reflected by H&E staining, and demyelination level, reflected by LFB staining, of the spinal cord; **(E)** H&E staining showed extensive inflammation in the spinal cord of NMOSD mice. LFB staining depicted demyelination in the spinal cord of NMOSD mice. Scale bar=200µm. **(F, G)** Statistics of immune cell infiltration, reflected by H&E staining, and demyelination level, reflected by LFB staining, in the brain. Scale bar=200µm. The experiment was repeated twice, with similar results. Data were presented as the mean±SEM; *P < 0.05 vs control group; n=6 in each group. LSD-t test was used.

Scores of the EAE mice peaked on Day 17 p.i. (**Figure 3A**). Before BBB opening and NMO-IgG injection, the clinical scores of mice in the EAE, EAE+BBB and NMOSD groups did not reveal any difference. After the injection of NMO-IgG and complement, mice in the NMOSD group suffered more severe symptoms of paralysis. Significantly higher scores were shown in mice in the NMOSD group than in mice in the EAE and EAE+BBB groups. There was no significant difference between mice in the EAE and EAE+BBB groups.

### Marked Inflammation and Demyelination in the NMOSD Group

Mice were sacrificed on Day 24 p.i. Brains, spinal cords and optic nerves were harvested. H&E staining was performed to evaluate the level of inflammatory infiltration. Compared to mice in the EAE and EAE + BBB groups, after the injection of NMO-IgG and human complement, mice in the NMOSD group presented more marked inflammation in the spinal cord and optic nerve (**Figures 3B–E**). However, no evident inflammation was found in the brains of the 4 groups of mice (**Figures 3F, G**). The results suggest that with the help of low frequency ultrasound, NMO-IgG could cause more severe optic neuritis and myelitis.

Consistent with the inflammation, the injection of 100 μg of NMO-IgG and 100 μL of human complement from patients also produced extensive demyelination in the optic nerve and spinal cord (**Figures 3B–E**). Although demyelination could be found in the spinal cords in the EAE and EAE + BBB groups, it was more extensive in the NMOSD group. It is worth noting that there was little demyelination in the optic nerves of the EAE and EAE+BBB groups, while optic nerves in the NMOSD group showed demyelination to some extent, which is a particular characteristic of the NMOSD model. No or little demyelination in the brain was observed in the 4 groups (**Figures 3F, G**). These results suggest that in the presence of additionally compromised integrity of the BBB caused by low frequency ultrasound, NMO-IgG could cause more severe demyelination in the optic nerve and spinal cords.

**Figure 4:**
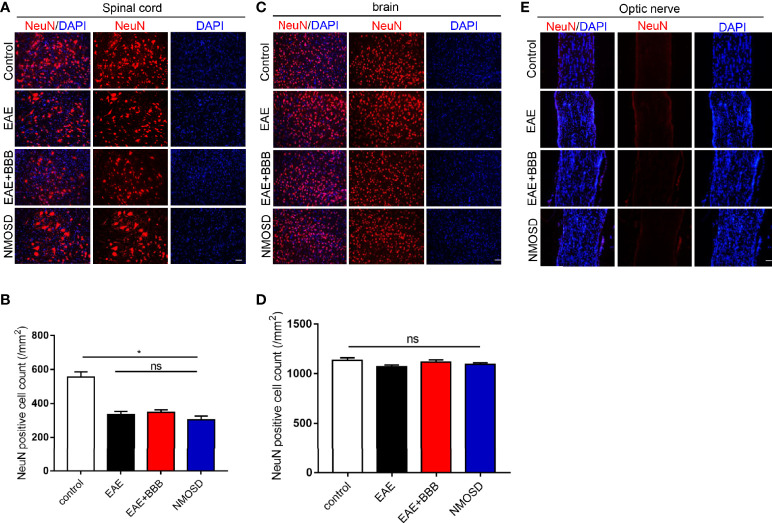
NeuN expression in the NMOSD model **(A, B)** Loss of NeuN positive cells in the spinal cord and statistic results, scale bar=50µm; **(C, D)** The number of NeuN positive cells in the brain and statistic results, scale bar=50μm; **(E)** NeuN expression in the optic nerve. Data were presented as the mean±SEM; *P < 0.05 vs control group; n=6 in each group. LSD-t test was used. NS, not significant.

Although the NMOSD group showed a significant loss of NeuN+ cells compared with the control group in the spinal cord (**Figure 4A**), there was no significant difference of NeuN+ cells between EAE, EAE+BBB and NMOSD groups (**Figure 4B**). In addition, no evident loss of NeuN+ cells were found in the brain of the NMOSD group compared to other groups (**Figures 4C, D**). No NeuN+cells were found in the optic nerve of control, EAE, EAE+BBB and NMOSD groups (**Figure 4E**). These results suggest that NMO-IgG do not cause further loss of neurons in the NMOSD mice.

### Loss of AQP4 and GFAP Expression in the NMOSD Group

**Figure 5:**
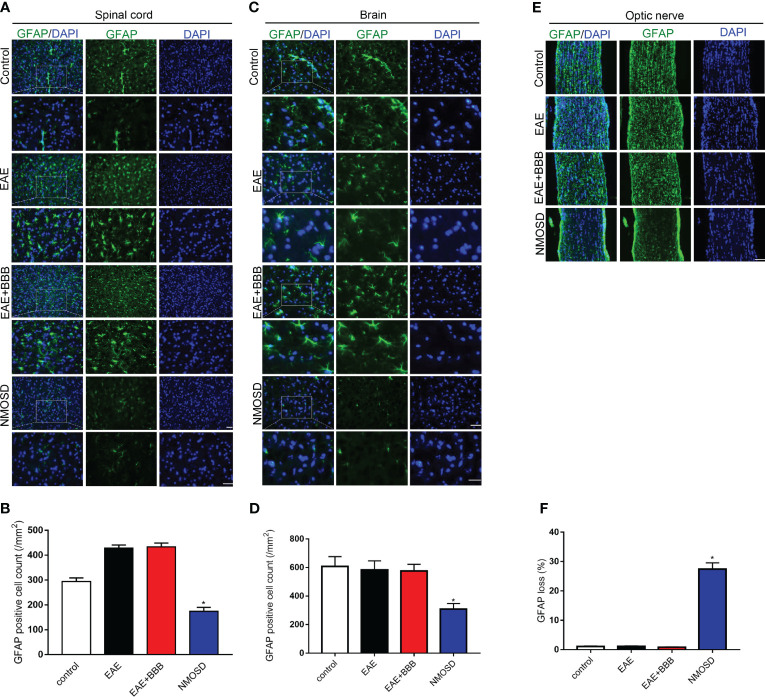
Loss of GFAP expression in the NMOSD model **(A, B)** Loss of GFAP expression in the spinal cord and statistic results, scale bar=50μm; **(C, D)** Loss of GFAP expression in the brain and statistic results, scale bar=50μm; **(E, F)** Loss of GFAP expression in the optic nerve and statistic results, scale bar=50μm; Scale bar=50µm. The experiment was repeated twice, with similar results. Data were presented as the mean±SEM; *P < 0.05 vs control group; n=6 in each group. LSD-t test was used.

**Figure 6:**
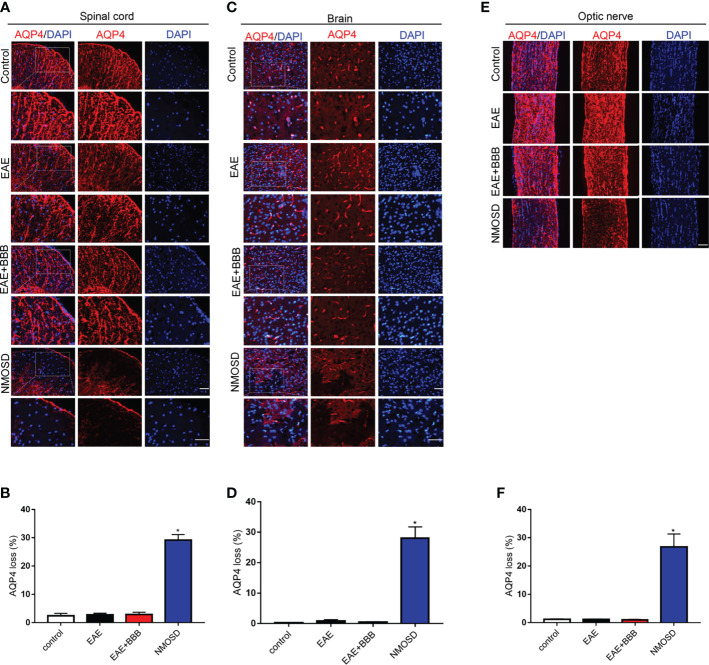
Loss of AQP4 expression in the NMOSD model **(A, B)** Loss of AQP4 expression in the spinal cord and statistic results, scale bar=50μm; **(C, D)** Loss of AQP4 expression in the brain and statistic results, scale bar=50μm; **(E, F)** Loss of AQP4 expression in the optic nerve and statistic results, scale bar=50μm; The experiment was repeated twice, with similar results. Data were presented as the mean±SEM; *P < 0.05 vs control group; n=6 in each group. LSD-t test was used.

GFAP (**Figure 5**) and AQP4 (**Figure 6**) expression in the optic nerve, spinal cord and brain were detected on day 24 p.i. In NMOSD mice, prominent AQP4 loss was noted in the spinal cord, brain and optic nerves, and AQP4 defects were characterized by massive astrocyte loss, which was reflected by GFAP loss. Accumulative fluorescence quantification analysis for each section revealed significantly decreased AQP4 and GFAP intensity compared with the other 3 groups. In mice of the EAE and EAE+BBB groups, no AQP4 or GFAP loss was found. The results suggest that NMO-IgG results in specific pathologic changes that are different from EAE.

### Human IgG Deposition in the NMOSD Group

**Figure 7:**
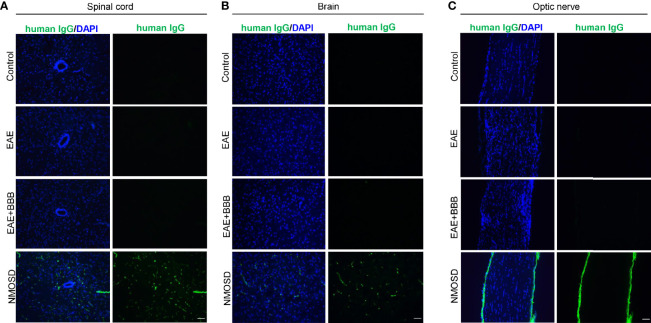
Human IgG deposition after NMO-lgG injection in the NMOSD model. NMOSD group showed human lgG deposition in the spinal cord **(A)**, brain **(B)** and optic nerve **(C)**. Scale bar=50μm.

To verify human NMO-IgG deposition in mouse tissue after NMO-IgG injection, we also detected human antibodies. In NMOSD mice, we found human IgG deposition in the brain, spinal cord and optic nerve (**Figure 7**). In the other 3 groups, we found no human IgG expression.

### MRI Revealed NMOSD-Like Optic Neuritis and Myelitis

**Figure 8:**
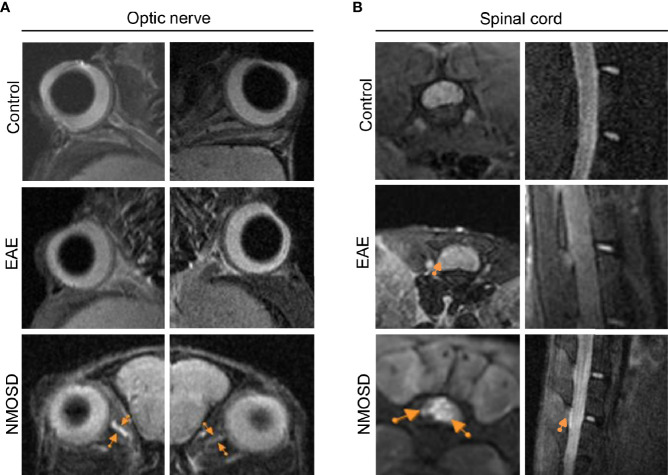
MRI presentation in the NMOSD model. **(A)** Both sides of the optic nerve in the NMOSD model showed increased intensity (arrows) on T2 FLAIR imaging, especially on the right side; **(B)** Increased intensity (arrows) was found in both transverse and longitudinal images of the spinal cord.

7-T small-animal MRI was performed on mice in the NMOSD group to further confirm optic neuritis and myelitis. Both sides of the optic nerves, especially the right optic nerve, showed hyperintensity on FLAIR sequences (**Figure 8A**). Increased intensity was also observed in both transverse and longitudinal images of the spinal cord (**Figure 8B**). In the EAE mice, MRI presented swelling but no high signals in both sides of optic nerves. In addition, some high signals were found in the peripheral of the spinal cord of EAE mice. No lesion was found in the optic nerve and spinal cord of control mice.

### Eye Segregation Defects in the dLGN of NMOSD Mice

**Figure 9:**
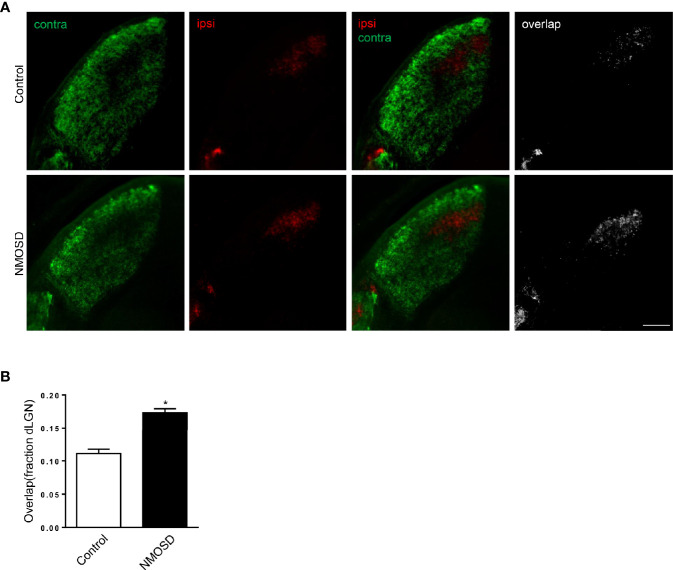
Eye-specific segregation in the NMOSD model. **(A)** An increased fraction of the dLGN in NMOSD mice was covered by ipsilateral RGC afferents, causing an increase in the overlap between ipsilateral and contralateral projections; **(B)** Representative images of eye-specific segregation defects. Scale bar=100 µm. The experiment was repeated twice, with similar results. Data were presented as the mean±SEM; *P < 0.05 vs control group; n=6 in each group. Unpaired t test was used.

Binocular inputs to the dorsal lateral geniculate nucleus (dLGN) overlap before remodeling to occupy eye-specific territories. During refinement, bilateral RGC output plays critical roles in visual circuit development by instructing the remodeling of retinofugal axon terminals in a retinotopic and eye-specific fashion (18). When one side of the visual input is impaired, the pattern of eye segregation changes. Therefore, we studied the area of ipsilateral and contralateral projections from RGCs in the dLGN to confirm the integrity of the visual pathway. Compared to the control group, the NMOSD group showed an increased area of overlap between the ipsilateral and contralateral projections in the dLGN. We noticed that the increased overlap mainly resulted from the abnormal expansion of ipsilateral projections (**Figures 9A, B**). The fraction of the dLGN covered by contralateral projections in the NMOSD mice showed no significant changes. These results suggest that there were eye segregation defects caused by abnormal RGC electrical activity.

## Discussion

Animal models of NMOSD were mainly constructed through systemic delivery of NMO-IgG or direct injection. Previously, researchers developed animal models of NMOSD through passive transfer of NMO-IgG into EAE mice/rats (19, 20). Harleen Saini et al. reported that NMO-IgG exacerbated the clinical course of EAE mice and induced NMOSD-like lesions including AQP4 and GFAP loss in the spinal cord, without observing the related pathologic changes in the optic nerve and brain. Notably, they tested the long-term effect of NMO-IgG and found that the worse clinical outcome in NMOSD group was maintained for as long as 40 days after the last injection of NMO-IgG. In addition, marked infiltration of granulocytes was found in the NMOSD mice while it is difficult to find neutrophil or eosinophils infiltration in EAE mice. However, this model of NMOSD is limited by the huge demand for NMO-IgG. In contrast, with the help of low-frequency ultrasound, we were able to induce the pathogenesis of NMOSD with a relatively lower amount of NMO-IgG. In the current study, further opening the BBB with low-frequency ultrasound may have promoted more NMO-IgG to enter the central nervous system.

A direct injection model has been established by a series of researchers (21–24). NMO-IgG from patients was directly injected into the mouse brain or eye. NMOSD-like lesions, including AQP4 expression loss, astrocytic swelling, perivascular complement deposition, and granulocytic infiltration, were observed after NMO-IgG was injected with human (but not mouse) complement (25). However, due in part to the limited diffusion of AQP4-IgG and complement from the injection site, the pathological effects in this model were confined to a small region around the injection site, and the injury caused by the direct injection may confuse the evaluation of clinical outcomes. Another limitation of these models is that the induction process is different from that in human NMOSD, in which NMO-IgG in the peripheral blood instead of NMO-IgG in the injected site initiates the pathology. In our model, NMO-IgG was injected intravenously and entered the central nervous system with the help of low-frequency ultrasound, reproducing the entry of NMO-IgG into the central nervous system. In addition, and the pathological changes found in the brain, spinal cord and optic nerve reproduced the diffuse extensive lesion typically found in NMOSD patients. In addition, low-frequency ultrasound caused BBB opening without any additional underlying injury, avoiding the problems posed by direct injection.

In the present study, the NMOSD group demonstrated a loss of astrocytic proteins in the brain, spinal cord and optic nerve, which was not found in the EAE and EAE+BBB groups. In addition, NMOSD mice presented more severe inflammation and demyelination in the spinal cord and optic nerve. However, there was no significant loss of NeuN+ cells in the NMOSD group, which is consistent with the previous finding that NMOSD mice had patchy demyelination and mild neutrophil infiltration but no neuronal cytotoxicity (26). We infer that neurons do not express AQP4, so they may not be directly attacked by NMO-IgG. It is plausible to infer that the higher clinical score in the NMOSD model is associated with an additionally decreased BBB integrity, as low-frequency ultrasound caused a lower expression level of tight junction protein (occludin and claudin-5) in the NMOSD than in the control and EAE group.

The optic neuritis and myelitis in NMOSD mice were further confirmed by 7-T MRI. At present, MRI is an important tool for the diagnosis of NMOSD patients. It has also been applied in EAE models of mouse, rat and non-human privates as well as some other CNS demyelination models to study their relevance to human disease (9–13). In our study, there was hyperintensity on FLAIR sequences in the spinal cord and optic nerve of NMOSD mice, which was not found in the control mice. Notably, hyperintensity was mainly found in the center of the spinal cord, which is similar to the radiological feature of NMOSD patients (27).

Optic neuritis in NMOSD patients may result in permanent loss of vision. In human optic nerves, AQP4 expression is enriched in astrocytic endfoot membrane domains (28). Previous studies of NMOSD animal models lack observations in optic nerves (5, 29). In our NMOSD mouse model, optic neuritis was observed with an incidence of 67/102 and was assessed extensively by histopathology and radiology. Actually, optic neuritis is not a unique marker for a NMOSD model. High rates of optic neuritis with immune cell infiltrations and demyelination were also observed in MOG-induced EAE mice (30–32). In our study, the incidence of optic neuritis in NMOSD mice was comparable with EAE mice, but the extent of inflammation and demyelination was more severe in NMOSD mice. Another feature of NMOSD mice is that AQP4 and GFAP loss in optic nerve, which is not found in EAE mice.

Moreover, we found eye segregation defects in the dLGN in the NMOSD model, which have not been reported in other NMOSD models. These defects might be caused by abnormal electrical RGC activity (16, 33, 34). Our results suggest that eye segregation defects were caused by abnormal RGC electrical activity in NMO-IgG-induced dysfunction, indicating that one critical cause of visual dysfunction in NMOSD might be abnormal visual information processing. Only the expansion of ipsilateral projection was found, while no reduction of the contralateral projections was noted. We infer that this change may be the early manifestation of visual pathway damage, and if the damage persists the projection from the other side would expand.

In conclusion, taking advantage of low-frequency ultrasound, we developed a mouse NMOSD model requiring a significantly lower dose of NMO-IgG and relatively simple equipment. We compared the pathologic changes in EAE mice. This model simulates the core features of NMOSD better with stable optic neuritis and myelitis with a high incidence. The pathological changes were further confirmed by radiological changes. Moreover, this novel method combining BBB opening with NMO-IgG could be applied to other animal models of neuroimmune diseases mediated by autoantibodies.

## Data Availability Statement

The original contributions presented in the study are included in the article/supplementary material. Further inquiries can be directed to the corresponding author.

## Ethics Statement

The studies involving human participants were reviewed and approved by Ethics Committee of the Ren Ji Hospital Affiliated to Shanghai Jiaotong University School of Medicine. The patients/participants provided their written informed consent to participate in this study. The animal study was reviewed and approved by animal care and use committees of Ren Ji Hospital Affiliated to Shanghai Jiaotong University School of Medicine.

## Author Contributions

WX, CX, and JL conceived, designed, and performed all experiments and drafted the manuscript. WZ gave suggestions on experimental design and methodology. XZ gave assistance in MRI scanning. HY, YC, JD, YW, YH, and YZ worked on IgG preparation and purification. YG conceived the study and revised the manuscript. All authors agreed the data and the final manuscript. All authors contributed to the article and approved the submitted version.

## Funding

This work was supported by the National Natural Science Foundation of China (82071341, 81801195, 81771295), Innovative research team of high-level local universities in Shanghai, Health and Family Planning Commission foundation of Shanghai (20194Y0087).

## Conflict of Interest

The authors declare that the research was conducted in the absence of any commercial or financial relationships that could be construed as a potential conflict of interest.

## Publisher’s Note

All claims expressed in this article are solely those of the authors and do not necessarily represent those of their affiliated organizations, or those of the publisher, the editors and the reviewers. Any product that may be evaluated in this article, or claim that may be made by its manufacturer, is not guaranteed or endorsed by the publisher.
