# Corrigendum: Low Frequency Ultrasound With Injection of NMO-IgG and Complement Produces Lesions Different From Experimental Autoimmune Encephalomyelitis Mice

**DOI:** 10.3389/fimmu.2022.842300

**Published:** 2022-01-25

**Authors:** Weiwei Xiang, Chong Xie, Jiaying Luo, Wei Zhang, Xinxin Zhao, Hong Yang, Yu Cai, Jie Ding, Yishu Wang, Yong Hao, Ying Zhang, Yangtai Guan

**Affiliations:** ^1^ Department of Neurology, Ren Ji Hospital, Shanghai Jiao Tong University School of Medicine, Shanghai, China; ^2^ Department of Ultrasound in Medicine, Shanghai Jiao Tong University Affiliated Sixth People’s Hospital, Shanghai, China; ^3^ Department of Radiology, Ren Ji Hospital, Shanghai Jiao Tong University School of Medicine, Shanghai, China; ^4^ Department of Neurology, The First Rehabilitation Hospital of Shanghai, Tongji University School of Medicine, Shanghai, China

**Keywords:** neuromyelitis optica, mouse, Aquaporin-4, blood-brain barrier, low-frequency ultrasound

In the original article, there was a mistake in **Figures 5** and **6** as published. The figures in their previous form do not best reflect the results. The corrected **Figures 5** and **6** appear below.

**Figure 5:**
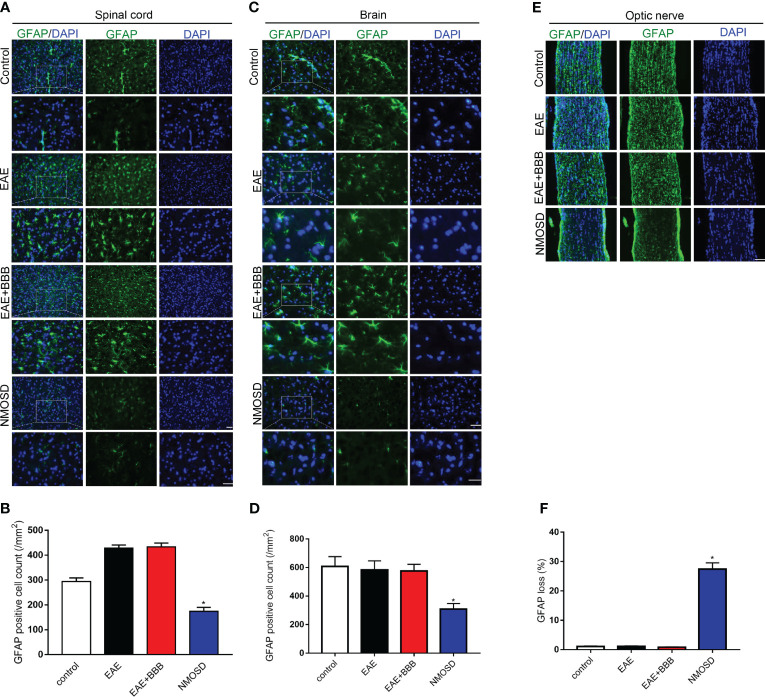
Loss of GFAP expression in the NMOSD model **(A, B)** Loss of GFAP expression in the spinal cord and statistic results, scale bar = 50μm; **(C, D)** Loss of GFAP expression in the brain and statistic results, scale bar = 50μm; **(E, F)** Loss of GFAP expression in the optic nerve and statistic results, scale bar = 50μm; Scale bar = 50μm. The experiment was repeated twice, with similar results. Data were presented as the mean ± SEM; *P < 0.05 vs control group; n = 6 in each group. LSD-t test was used.

**Figure 6:**
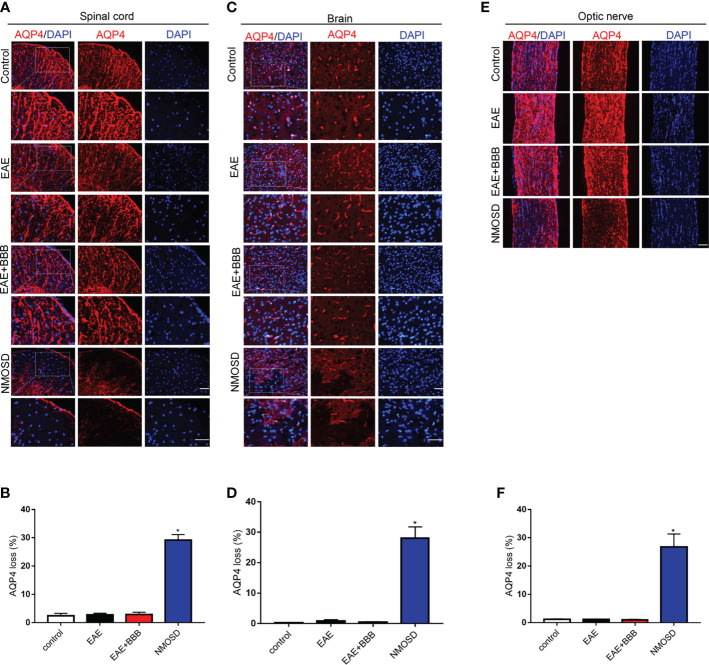
Loss of AQP4 expression in the NMOSD model **(A, B)** Loss of AQP4 expression in the spinal cord and statistic results, scale bar = 50μm; **(C, D)** Loss of AQP4 expression in the brain and statistic results, scale bar = 50μm; **(E, F)** Loss of AQP4 expression in the optic nerve and statistic results, scale bar = 50μm; The experiment was repeated twice, with similar results. Data were presented as the mean ± SEM; *P < 0.05 vs control group; n = 6 in each group. LSD-t test was used.

The authors apologize for this error and state that this does not change the scientific conclusions of the article in any way. The original article has been updated.

## Publisher’s Note

All claims expressed in this article are solely those of the authors and do not necessarily represent those of their affiliated organizations, or those of the publisher, the editors and the reviewers. Any product that may be evaluated in this article, or claim that may be made by its manufacturer, is not guaranteed or endorsed by the publisher.

